# Household income and academic performance in Korean adolescents: A longitudinal test of dual investment pathways

**DOI:** 10.1371/journal.pone.0353476

**Published:** 2026-07-09

**Authors:** Ran Kang

**Affiliations:** 1 Department of Child Psychology and Education, Sungkyunkwan University, Seoul, Republic of Korea; 2 Social Innovation Convergence Program, Sungkyunkwan University, Seoul, Republic of Korea; Universidade Federal do Tocantins, BRAZIL

## Abstract

Building on the Family Investment Model (FIM), the present study examined how household income shapes adolescents’ academic performance through material and relational investments within the Korean educational context. Drawing on three waves of data from the nationally representative Korean Children and Youth Panel Survey 2018 (N = 2,387), we estimated a longitudinal parallel mediation model that accounted for baseline levels of each construct and demographic covariates. Results indicated that household income predicted higher private educational expenditure but was not associated with parent–child communication. Indirect effects revealed that household income influenced academic performance through material investment in private education, whereas the relational pathway was not significant. However, the difference between the two indirect pathways did not reach statistical significance. These findings suggest that private educational expenditure may represent one important pathway linking family economic resources to adolescents’ perceived academic performance in the Korean educational context. These findings underscore how household economic resources are differentially converted into educational advantages through family investment processes, highlighting implications for understanding income-based educational stratification in highly competitive educational contexts.

## Introduction

Socioeconomic inequalities in children’s development remain a defining feature of contemporary family life across diverse cultural and national contexts. As family structures, parental work conditions, and broader socioeconomic environments have undergone rapid transformation in recent decades, the daily ecological contexts in which children grow have become increasingly stratified [1–2]. These structural shifts have heightened the salience of family economic resources, particularly household income, which shapes children’s academic and socioemotional development through its influence on the material and relational resources available within the home [3–6]. Against this backdrop, understanding how family income is translated into children’s outcomes through family economic and investment processes has become a central focus in family research.

A long-standing body of developmental scholarship demonstrates that income-based differences in cognitive stimulation, access to learning opportunities, and quality of relational environments contribute to enduring disparities in academic performance and long-term educational attainment [7–12]. Yet, despite decades of evidence linking economic resources to child well-being, far less attention has been devoted to unpacking the specific mechanisms through which income shapes developmental trajectories—and to evaluating whether these pathways operate similarly across distinct sociocultural contexts. Addressing these gaps requires an explicit focus on the family processes through which income is invested in children’s lives, thereby laying the groundwork for a nuanced understanding of how socioeconomic resources become converted into developmental advantage.

### The Family Investment Model

The Family Investment Model (FIM) [13–14] provides a central framework for understanding how family economic resources shape children’s development by enabling access to enriched learning environments and educational opportunities. Building on this parental investment framework [13], FIM conceptualizes income as a foundational resource that parents convert into educational opportunities, enriched home environments, and supportive parent–child interactions. Subsequent research extended this economically rooted framework into a developmentally informed model of family processes [14,15].

According to FIM, parents’ economic resources support two broad types of investments: material investments—such as educational expenditures and cognitively stimulating materials—and relational investments, including supportive parenting practices and parent–child interactions. Subsequent developmental research refined early economic formulations by empirically distinguishing these pathways, with Yeung and colleagues [14] providing one of the first clear distinctions between material and relational investments. Although this dual-pathway structure is now widely recognized, most empirical evidence has been drawn from Western contexts, underscoring the importance of evaluating how these processes operate in distinct sociocultural settings such as Korea. At its core, the Family Investment Model assumes that family income influences children’s developmental outcomes through two analytically distinct yet complementary pathways—material and relational investments.

Parents’ economic resources shape children’s development in part through the learning environments they are able to provide. Families with greater financial resources can offer cognitively stimulating materials, high-quality learning opportunities, and structured activities that promote academic growth [16–18]. These material investments, particularly direct educational expenditures, have been consistently linked to gains in academic skills, especially in contexts where access to enriched educational resources is stratified by socioeconomic status [3,19]. Children from higher-income households also benefit from supplemental learning experiences that directly support academic performance [20,21], underscoring the role of material investments in transmitting family income into adolescents’ achievement [22].

Socioeconomic resources further influence the quality of home learning environments. Higher-income parents tend to provide more stimulating materials, richer communication, and cognitively enriching experiences that contribute to early literacy and vocabulary development [18,23]. International evidence—including studies from Russia and the UK—demonstrates that SES differences in cognitive development largely reflect disparities in parental inputs and early learning opportunities [24,25]. Within-family analyses also show that increases in household income are followed by improvements in home learning conditions [26]. Research in Korea aligns with these patterns, indicating that early socioeconomic adversity continues to shape academic trajectories into adolescence and adulthood [27].

In addition to material investments, a substantial body of research highlights the relational mechanisms through which economic resources shape youth development. Income-based advantages allow parents to engage in more frequent, emotionally supportive, and developmentally responsive interactions with their children—central elements of the relational investment pathway in FIM. Higher-income parents tend to communicate more often and provide conversational scaffolding within warm relational climates that foster motivation, self-regulation, and academic engagement [28–30].

Parent–child communication, as one observable dimension of relational investment, has been identified as a key relational mechanism linking socioeconomic resources to academic outcomes. Richer verbal exchanges are associated with stronger literacy skills, improved school adjustment, and higher academic performance [31,32]. Conversely, socioeconomic hardship can constrain parents’ capacity to sustain high-quality engagement, contributing to reduced academic involvement among adolescents [33]. Evidence from Korea aligns with this relational pathway. Previous research has shown that socioeconomic resources predict adolescents’ academic performance through structured parental involvement and adolescents’ time use, illustrating how socioeconomic advantages shape relational and behavioral processes within families [34]. Together, these findings indicate that socioeconomic conditions shape opportunities for supportive communication and relational climates that facilitate adolescents’ academic adjustment.

Recent research has refined the distinction between material and relational investments and demonstrated that these pathways can operate simultaneously yet differentially across developmental domains. Previous research has shown that income predicts children’s cognitive readiness through enriched learning environments—classic material pathways—whereas parents’ emotional well-being and parenting behaviors shape other key developmental processes [35,36]. Extending this line of work, educational inputs and socioemotional family processes have been shown to function as analytically separable mechanisms linking socioeconomic conditions to school transitions [28]. Further research has shown that socioeconomic advantage is associated with systematic differences in how parents allocate time and engagement with their children, underscoring that material and relational investments do not respond uniformly to changes in family resources [15].

Consistent with this evidence, longitudinal studies across diverse regions increasingly conceptualize SES–achievement associations as dual, partially independent pathways comprising material and relational investments [22,37]. However, although much of the foundational FIM research has emphasized early childhood, relatively little is known about how these pathways operate during later developmental periods, when academic demands intensify and achievement gaps become more pronounced [38]. This gap highlights the importance of examining whether FIM’s dual pathways extend to older children navigating increasingly complex academic and familial environments.

### Material and Relational Investments in the Korean Context

Korea represents a distinctive sociocultural environment in which FIM’s dual pathways may be particularly salient. Confucian familism places strong emphasis on parental responsibility for children’s academic success [39], motivating families to invest heavily in both material and relational forms of support. Korea’s educational landscape is marked by intense academic pressure, extensive participation in private academies, and substantial household educational expenditure on supplemental instruction. In 2024 alone, households spent more than 29 trillion KRW on private education, with participation rates exceeding 80% across school levels [40]. As per-student expenditures continue to rise amid declining birth rates, SES-based disparities in access to learning opportunities remain pronounced. Families with greater economic resources are thus better positioned to invest in tutoring, structured learning activities, and individualized academic guidance—conditions that make Korea an especially compelling context for examining SES-linked differences in material and relational investments.

Large-scale Korean studies document clear socioeconomic gradients in family investment patterns. Higher-income families spend substantially more on private education, and private‐education expenditures consistently predict children’s academic performance [41]. Other nationally representative evidence shows that income shapes both material investments—such as private education expenditures—and relational investments, including parent–child communication and emotional support [42]. National survey data further reveal limited daily parent–child communication in Korea: approximately 61% of adolescents report talking with their parents for less than one hour per day, with reduced communication linked to lower emotional connectedness and family cohesion. These findings align with the relational pathway delineated within FIM [30,31]. Finally, OECD and KEDI reports show that Korean households devote unusually large proportions of disposable income to private education [43,44], reinforcing Korea as a setting where SES‐based disparities in both investment types are particularly strong and highly relevant for testing FIM’s dual pathways.

### Current Study

Despite substantial evidence supporting dual-pathway processes within the Family Investment Model (FIM), relatively few studies have employed multi-wave longitudinal mediation designs that adjust for baseline levels of both mediators and perceived academic performance. Such limitations restrict the ability to draw temporal inferences about how these two investment pathways unfold over time. Moreover, much of the Korean literature has relied on cross-sectional or single-pathway approaches that do not simultaneously estimate the independent and comparative contributions of material and relational investments. As a result, important questions remain regarding whether family income exerts stronger effects through one pathway than the other and whether these processes remain robust after accounting for prior levels of investment and achievement.

To address these gaps, the present study applies a three-wave parallel mediation model to examine how household income predicts adolescents’ perceived academic performance through material investment (private educational expenditure) and relational investment (parent–child communication). This approach enables a rigorous test of pathway-specific effects within Korea’s distinctive educational context, where material and relational resources carry substantial implications for income-based educational inequality among school-age youth.

**Fig 1:**
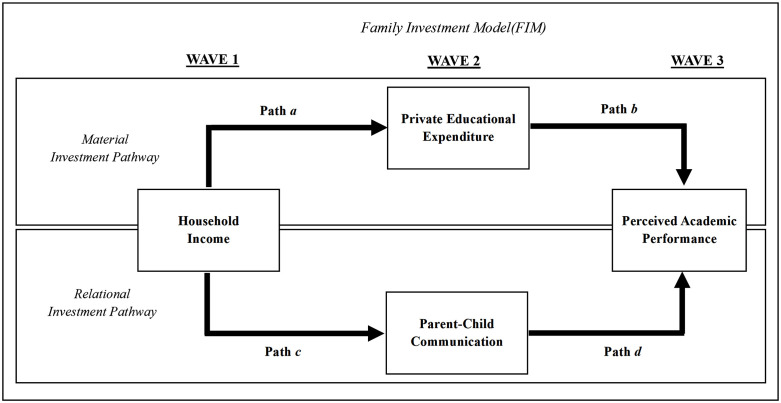
Proposed Longitudinal Parallel Mediation Model based on FIM.

Drawing on the Family Investment Model and prior research, four hypotheses were formulated for this study. Fig 1 presents the conceptual model. First, higher household income at Wave 1 was expected to predict higher levels of material investment (private educational expenditure) and relational investment (parent–child communication) at Wave 2 (Hypothesis 1). In turn, material and relational investments at Wave 2 were expected to predict higher perceived academic performance at Wave 3, controlling for baseline achievement (Hypothesis 2). Next, household income was expected to show indirect associations with perceived academic performance through (a) material investment and (b) relational investment (Hypothesis 3). Finally, the relative magnitudes of the two indirect pathways were examined exploratorily, with no specific directional hypothesis regarding which pathway would be stronger (Hypothesis 4).

## Materials and methods

### Sample

The present study used data from the Korean Children and Youth Panel Survey 2018 (KCYPS 2018), a nationally representative longitudinal study conducted by the National Youth Policy Institute (NYPI). The KCYPS 2018 consists of two grade-based cohorts, and the current study utilized the Grade 4 cohort, which includes early adolescents recruited from elementary schools using a stratified, multi-stage cluster sampling design. Early adolescence represents a developmentally sensitive period during which academic motivation, cognitive strategies, and parent–child interactions begin to undergo meaningful transitions, making the Grade 4 cohort an appropriate starting point for examining longitudinal changes in family investment processes.Wave 1 was collected in 2018 when participants were in the fourth grade, and follow-up surveys were conducted annually. Data were obtained from adolescents and their parents through self-report questionnaires.

A total of 2,607 early adolescents (50.4% male) participated at Wave 1 (Mage = 10.00, SD = 0.10). Among them, 2,411 remained in the panel by Wave 3, yielding a retention rate of 92.5%. After excluding cases with missing values on key study variables, 2,387 adolescents were included in the final analytic sample (based on Mplus model estimation results). The average monthly household income was 512.16 (unit = 10,000 KRW), corresponding to approximately 5,121,600 KRW per month. The original data collection procedures for KCYPS 2018 were reviewed and approved by the relevant Institutional Review Board and national statistical authorities. The present study used publicly available, de-identified secondary data from KCYPS 2018. The dataset contained no personally identifiable information, and the authors had no direct contact with participants. Because the study involved secondary analysis of existing anonymized data, no institutional review board approval or exemption determination was obtained for the present study.

### Measures

#### Household income.

Household income was measured at Wave 1 (2018) using a parent-reported item assessing the family’s average monthly income. Parents selected one of 12 ordered response categories ranging from *“no income”* to *“10 million KRW or more.”* The original categorical responses were converted into midpoint values (unit = 10,000 KRW) to approximate continuous monthly household income. Because the highest category was open-ended, a value of 11 million KRW was assigned by extending the width of the adjacent income interval (9–10 million KRW), corresponding to the midpoint of a hypothetical 10–12 million KRW range. The resulting variable was used as a continuous indicator of household economic resources, with higher scores reflecting greater monthly income.

#### Private educational expenditure.

Private educational expenditure was measured at Wave 2 (2019) using a parent-reported item assessing the family’s average monthly spending on private education (unit = 10,000 KRW). Parents indicated whether the adolescent received any private education and, if so, reported the monthly amount spent. Adolescents who did not participate in private education were assigned a value of zero expenditure. These items were adapted and refined from corresponding measures used in the Korean Children and Youth Panel Survey 2010 (KCYPS 2010) [45]. To account for positive skewness, private educational expenditure was log-transformed using log10(x + 1) prior to analysis. The addition of 1 allowed the inclusion of cases reporting no private educational expenditure. The log-transformed variable was subsequently used as a continuous indicator of material investment, with higher values representing greater monthly spending.

#### Parent–child communication.

Parent–child communication was measured at Wave 2 (2019) using two items assessing the average amount of time adolescents spent talking with their parents on weekdays and weekends. According to the original KCYPS measurement documentation [45], the parent–child communication measure was developed as part of the family environment domain intended to capture parent–child interaction and adolescents’ developmental contexts. Adolescents reported their communication time using seven ordered categories ranging from *“none”* to *“4 hours or more.”* These items were adapted and refined from measures used in the 2013 Youth Media Use Survey [46], conducted by the Ministry of Gender Equality and Family. A composite score was computed by averaging the weekday and weekend communication items, with higher values indicating more frequent parent–child communication.

#### Perceived academic performance.

Adolescents’ perceived academic performance was measured at Wave 3 (2020) using a single self-report item assessing their overall academic standing during the previous semester. Adolescents rated their academic performance on a five-point scale ranging from 1 (*“very poor”*) to 5 (*“very good”*). The original survey also included a sixth response option (*“don’t know”*), which was treated as missing and excluded from analyses. Following common practices in Korean panel studies, this item was adapted and refined from the academic performance measure used in the Korean Children and Youth Panel Survey 2010 (KCYPS 2010) [45]. Higher scores reflected higher levels of perceived academic performance.

#### Covariates.

Three demographic covariates were included in all models to account for background heterogeneity among families: adolescent gender, adolescent age, and parental education. Adolescent gender was recoded so that 0 = male and 1 = female. Adolescent age (Wave 1) was treated as a continuous variable. Parental education was included as a covariate to account for parental human capital resources beyond household income. Although household income and parental education are both indicators of family socioeconomic background, they capture distinct dimensions of family resources [47]. Parental education was measured using parent-reported educational attainment for the father, mother, and guardian. Responses coded as “don’t know” or “not applicable” were treated as missing. Rather than averaging or summing across parents, parental education was operationalized as the highest educational attainment reported among the father, mother, or guardian, with higher scores indicating higher levels of parental education.

These covariates (gender, age, and parental education) were specified as exogenous predictors of both Wave 2 mediators—private education expenditure and parent–child communication—and the Wave 3 outcome, perceived academic performance. In addition, prior levels of the focal constructs (Wave 1 private education expenditure, parent–child communication, and perceived academic performance) were included as baseline controls in their respective equations.

### Statistical analysis

All analyses were conducted in Mplus 7.4 [48]. Prior to estimating the focal models, descriptive statistics and zero‐order correlations were examined for all study variables. To test the hypothesized Family Investment Model (FIM) pathways, a longitudinal path model with parallel mediators was estimated. In this model, Wave 1 household income was specified as the predictor, Wave 2 private educational expenditure and parent–child communication were specified as parallel mediators, and Wave 3 perceived academic performance served as the outcome. Wave 1 private educational expenditure, parent–child communication, and perceived academic performance were included as baseline controls to account for prior levels of the focal constructs. To isolate the unique effects of household income, adolescent gender, age, and parental education were included as covariates predicting both Wave 2 mediators and the Wave 3 outcome. The indirect effects of household income on perceived academic performance through each mediator were estimated using bias-corrected bootstrap procedures with 5,000 resamples. Both unstandardized coefficients (b) and standardized coefficients (β) were reported to facilitate interpretation of the magnitude and practical significance of the observed effects. The model also estimated the difference between the two specific indirect effects (income → private educational expenditure → perceived academic performance vs. income → parent–child communication → perceived academic performance).

Model fit was evaluated using multiple indicators, including the *χ²* goodness-of-fit, Comparative Fit Index (CFI ≥ .90), Tucker–Lewis Index (TLI ≥ .90), Root Mean Square Error of Approximation (RMSEA ≤ .08), and Standardized Root Mean Square Residual (SRMR ≤ .08) [49]. The analytic sample consisted of 2,387 adolescents. Missing data were handled using full information maximum likelihood (FIML), which utilizes all available information under the assumption that data are missing at random (MAR). All models were estimated using maximum likelihood (ML) estimation.

## Results

### Preliminary analysis

Sample characteristics are presented in Table 1. Means, standard deviations, and bivariate correlations among all study variables are presented in Table 2. Household income at Wave 1 was positively associated with both private educational expenditure and parent–child communication at Wave 2. In turn, both investment indicators assessed at Wave 2 were positively correlated with perceived academic performance at Wave 3. Baseline measures of private educational expenditure, parent–child communication, and perceived academic performance at Wave 1 were also significantly related to their Wave 2 or Wave 3 counterparts, demonstrating moderate rank-order stability across waves. All correlations were in the expected directions and consistent with the Family Investment Model. To examine potential attrition bias, participants included in the analytic sample were compared with those excluded because of missing data. No significant differences were observed in gender, age, or baseline academic performance. Although participants included in the analytic sample reported slightly higher levels of parental education, household income, parent–child communication, and private educational expenditure, the associated effect sizes were small (effect sizes ranged from *d* = .23 to *d* = .29). These findings suggest that attrition was unlikely to substantially bias the study findings.

**Table 1:** Characteristics of Participants.

Variable	Category	*n(*%)	M(SD)	Min	Max
Adolescent Variables					
Adolescent Gender	Male	1,313(50.4)			
	Female	1,294(49.6)			
Adolescent Age (W1)			10.00(0.10)	9.00	11.00
Parent Variables					
Father’s Education	Middle school or below	50(1.9)			
	High school	602(23.1)			
	College (2–3 yrs)	597(22.9)			
	University (4 yrs or above)	964(37.0)			
	Graduate school	282(10.8)			
Mother’s Education	Middle school or below	58(2.2%)			
	High school	693(26.6)			
	College (2–3 yrs)	754(28.9)			
	University (4 yrs or above)	850(32.6)			
	Graduate school	185(7.1)			
Household Income (unit = 10,000 KRW; ≈ $7.52)			512.16(229.31)	0.00	1100.00

Note. Values are based on all available Wave 1 observations from the 2018 Korean Children and Youth Panel Survey (KCYPS 2018). Sample sizes vary across variables because of item-level missing data. For reference, 2,607 adolescents and their parents participated at Wave 1, and 2,411 remained in the panel by Wave 3 (retention rate = 92.5%). The longitudinal mediation model was estimated using the final analytic sample (N = 2,387). Household income was converted to midpoint values and is reported in units of 10,000 KRW (South Korean won).

**Table 2:** Means, Standard Deviations, and Zero-Order Correlations among Study Variables.

Study Variable	1	2	3	4	5	6	7	*M*	*SD*
1. Household Income (W1)	1							512.16	229.31
2. Parent–Child Communication (W1)	.10^**^	1						4.29	1.56
3. Parent–Child Communication (W2)	.06^*^	.37^***^	1					4.27	1.42
4. Perceived Academic Performance (W1)	.17^***^	.15^***^	.09^**^	1				3.87	0.80
5. Perceived Academic Performance (W3)	.10^***^	.09^**^	.12^***^	.30^***^	1			3.72	0.80
6. Private Educational Expenditure (W1)	.36^***^	.07^**^	.06^**^	.18^***^	.12^***^	1		1.40	0.53
7. Private Educational Expenditure (W2)	.29^***^	.04^*^	.03	.16^***^	.14^***^	.58^***^	1	1.41	0.54

*Note.* W=Wave. ^*^p<.05, ^**^p<.01, ^***^p<.001.

### Direct effects of household income and family investments on perceived academic performance

**Fig 2:**
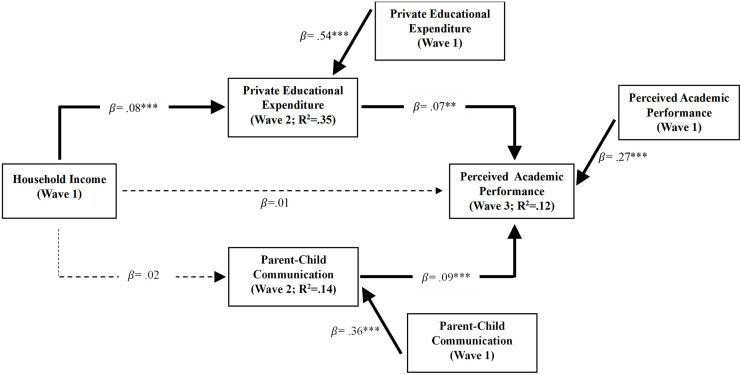
Longitudinal parallel mediation model of material and relational investment pathways based on the Family Investment Model. *Note***.** Standardized coefficients (β) are presented. Solid arrows indicate statistically significant paths, and dashed arrows indicate non-significant paths. The figure depicts a longitudinal parallel mediation model linking household income (W1), private educational expenditure and parent–child communication (W2), and perceived academic performance (W3), while controlling for prior levels of private educational expenditure, parent–child communication, and perceived academic performance. Covariates include adolescent gender, age, and parental education. ^*^p < .05. ^**^p < .01. ^***^p < .001.

The results regarding the direct paths from household income to family investments and perceived academic performance, as well as the direct effects of family investments on adolescents’ achievement, are presented in Fig 2. The longitudinal path model demonstrated an acceptable fit to the data, *χ²*(7) = 13.10; *p* = .070; CFI = .996; TLI = .987; RMSEA = .019 (90% CI [.000, .035]); SRMR = .008. These indices indicate that the hypothesized model adequately represented the observed data.

Consistent with Hypothesis 1, household income at Wave 1 significantly predicted higher levels of material investment at Wave 2 (*β* = .08, *p* < .001, 95% CI [.04, .11]). In contrast, household income was not significantly associated with relational investment, as measured by parent–child communication (*β* = .01, *p* = .468, 95% CI [–.03, .03]). Baseline levels of each construct demonstrated moderate rank-order stability over time: prior private educational expenditure predicted Wave 2 expenditure (*β* = .54, *p* < .001, 95% CI [.49, .58]), prior parent–child communication predicted Wave 2 communication (*β* = .36, *p* < .001, 95% CI [.29, .39]), and prior perceived academic performance predicted Wave 3 performance (*β* = .27, *p* < .001, 95% CI [.23, .30]).

Regarding Hypothesis 2, both investment indicators at Wave 2 significantly predicted perceived academic performance at Wave 3, above and beyond baseline achievement and demographic covariates. Private educational expenditure positively predicted later perceived academic performance (*β* = .07, *p* < .01, 95% CI [.03, .12]), and parent–child communication also exerted a positive effect (*β* = .09, *p* < .001, 95% CI [.05, .12]). Household income at Wave 1, however, was not directly associated with Wave 3 perceived academic performance (*β* = .01, *p* = .711, 95% CI [–.03, .05]), suggesting that income-related disparities in achievement operate primarily through indirect mechanisms rather than direct transmission. The R-squared values indicated that the model accounted for meaningful variance in each mediator and outcome: 34.8% of the variance in private educational expenditure, 13.9% in parent–child communication, and 11.5% in perceived academic performance.

### Parallel mediating effects of material and relational investments

The results regarding the mediational pathways from household income to adolescents’ perceived academic performance through material and relational investments are presented in Fig 2. Consistent with Hypothesis 3, the total indirect effect of household income on perceived academic performance through the two Wave 2 investment indicators was statistically significant (*β* = .007, *p* < .05, 95% CI [.002, .013]). Household income positively predicted private educational expenditure at Wave 2 (*β* = .077, *p* < .001), which in turn positively predicted perceived academic performance at Wave 3 (*b* = .118, *β* = .074, *p* < .01). Consequently, the material investment pathway was statistically significant (*β* = .006, *p* < .01, 95% CI [.002, .011]). In contrast, the relational investment pathway was not significant: household income (Wave 1) → parent–child communication (Wave 2) → perceived academic performance (Wave 3) (*β* = .001, *p* = .508, 95% CI [–.002, .004]).

As specified in Hypothesis 4, the present study exploratorily compared the relative magnitudes of the two indirect effects. This comparison indicated that the difference in magnitude did not reach statistical significance (Δ*β* = .002, *p* = .102, 95% CI [–.000, .004]). Although the difference between the two indirect effects was not statistically significant, an indirect pathway through private educational expenditure was observed, whereas an indirect pathway through parent–child communication was not observed. A robustness analysis using the original ordinal household income variable yielded substantively identical conclusions to those reported in the main analysis (see S1 Table). Additional robustness analyses using weekday and weekend communication separately likewise yielded substantively identical conclusions (see S2 Table).

## Discussion

The present study used a three-wave parallel mediation model to clarify how household income shapes adolescents’ educational outcomes through material and relational investments within the Family Investment Model (FIM) framework. Consistent with the primary mediation hypotheses, an indirect pathway through private educational expenditure was observed, whereas an indirect pathway through parent–child communication was not observed. This finding suggests that private educational expenditure may be one important pathway linking family economic resources to adolescents’ perceived academic performance in the Korean educational context. However, because the difference between the material and relational pathways was not statistically significant, caution is warranted when interpreting the relative importance of the two pathways. This study extends prior Family Investment Model research by applying a dual-pathway framework to later childhood in Korea—a life-course period in which parental investments intensify but remain understudied. By incorporating baseline levels of investment and achievement, the present study adds longitudinal evidence on how family investment processes relate to income-linked differences in academic outcomes.

Beyond identifying evidence for a material investment pathway, the present findings contribute to theoretical refinement of the Family Investment Model. Although decades of prior research have emphasized dual material and relational pathways, few studies have tested these processes using multi-wave designs with baseline controls. The current study demonstrates that once prior levels of investment and achievement are considered, an indirect effect through private educational expenditure remained statistically significant, whereas an indirect effect through parent–child communication did not. This divergence underscores the context-dependent nature of FIM processes and suggests that pathway strength may vary across developmental periods or sociocultural environments. By directly comparing the two pathways within a unified analytic framework, this study provides empirical evidence that mechanisms linking income to achievement are not uniform but instead exhibit systematic variation across contexts. These findings highlight the context-dependent nature of resource conversion processes within FIM, suggesting that the relative salience of investment pathways may vary meaningfully across sociocultural settings. More specifically, the present findings suggest that private educational expenditure may represent a particularly relevant pathway through which family economic resources are translated into adolescents’ perceived academic performance within the Korean educational system. At the same time, because the difference between the two indirect effects was not statistically significant, stronger conclusions regarding the relative importance of material and relational investments should be drawn cautiously. This pattern may reflect a context-specific configuration of FIM processes shaped by the institutionalization of private education and the central role of financial resources in educational attainment.

A possible explanation for the significant material investment pathway observed in the present study lies in the distinctive ways socioeconomic resources are converted into educational advantages in Korea. Prior research consistently shows that income differences in Korea are expressed most directly through private educational expenditure—an institutionalized form of material investment that is tightly coupled with academic outcomes. For example, higher-income households have been shown to allocate disproportionately greater resources to private education, suggesting that economic advantage translates into quantifiable and immediate learning opportunities [41].

Taken together, these findings suggest that in Korean educational contexts, where private education is highly institutionalized and strongly tied to academic trajectories, private educational expenditure may constitute a particularly visible mechanism through which family economic resources are translated into adolescents’ perceived academic performance. This pattern aligns with the present study’s results, in which only the material pathway significantly mediated the association between household income and perceived academic performance. Although relational investments such as parent–child communication remain important for adolescents’ socioemotional functioning, their influence on academic outcomes may be comparatively indirect or contingent on additional contextual factors.

Importantly, the present study also advances the Family Investment Model by addressing a critical developmental period that has been largely overlooked in prior research. Whereas most FIM applications focus on early childhood, parental investments in Korea intensify during the elementary school years, particularly as children move into upper grades and academic competition becomes increasingly institutionalized. For instance, national survey data indicate that private educational expenditure rises sharply beginning in the upper elementary years, reflecting heightened parental efforts to secure future academic advantage [40].

Notably, this period also marks a shift in parental involvement patterns, with families increasingly mobilizing both financial and relational resources to support school-related demands. Against this backdrop, the present study provides a meaningful extension of the FIM literature by applying a dual-pathway framework to children in the later elementary years—a developmental stage in which parental investments are especially concentrated yet understudied in the context of socioeconomic disparities. This focus highlights the importance of examining FIM processes beyond early childhood and underscores the relevance of socioeconomic resources during a period when academic trajectories begin to solidify. The present study, by modeling material and relational investments as parallel mediators, enabled a direct test of pathway-specific mechanisms—a particularly valuable contribution in a context where different forms of parental investment may vary in salience. Finally, the use of a large, nationally representative sample enhances the generalizability of these findings and underscores the importance of examining socioeconomic gradients in academic outcomes among Korean youth.

### Limitation

The present findings should be interpreted in light of several limitations. First, the longitudinal design helps clarify the temporal ordering among variables, but the study is still based on observational data, which limits strong causal interpretations. It is also possible that unmeasured factors may have influenced the observed associations. Second, the measures of private educational expenditure and parent–child communication capture only narrow facets of material and relational investments. Broader, multidimensional assessments of family investment processes may reveal more complex patterns. In particular, parent–child communication time may not fully capture the diverse ways in which relational investment is expressed within Korean families. Parental involvement and care may also be conveyed through instrumental support, educational monitoring, shared activities, and other forms of daily engagement that were not available in the present dataset. Future research would benefit from incorporating more comprehensive measures of parent–child relationships and family interaction processes to better capture culturally relevant forms of relational investment. In addition, the nonsignificant relational pathway observed in this study may reflect limitations related to measurement sensitivity or developmental timing, rather than the absence of meaningful relational processes. Third, the KCYPS 2018 dataset did not include measures of household wealth, assets, home ownership, or net worth. Therefore, the present analyses were unable to distinguish the effects of household income from broader forms of accumulated economic resources. Future research would benefit from incorporating more comprehensive indicators of family socioeconomic resources, including measures of household wealth, assets, home ownership, and intergenerational transfers, to better distinguish the unique contributions of income and wealth to family investment processes. Fourth, although the difference between the two indirect pathways was not statistically significant, this null finding may reflect limited statistical power to detect small effects rather than the absence of meaningful variation. Finally, adolescents’ perceived academic performance was assessed using a subjective single-item measure rather than objective academic records. Therefore, the findings should be interpreted as reflecting adolescents’ perceptions of their academic achievement, which may not fully capture their broader academic competencies or actual achievement levels.

### Implication and future direction

In addition to refining theoretical understanding of how material and relational pathways function within the FIM framework, the present findings carry meaningful implications for research and policy aimed at reducing socioeconomic disparities in academic outcomes. The finding that private educational expenditure emerged as the only significant mediator underscores the need for policies that reduce families’ dependence on private education or expand access to high-quality learning resources for lower-income youth. At the same time, the nonsignificant relational pathway highlights the need for future research to examine a broader set of relational investments, as communication reflects only one facet of parental relational engagement. Together, these implications emphasize that both structural interventions targeting educational inequalities and nuanced examinations of relational processes are essential for understanding how socioeconomic resources shape adolescents’ developmental trajectories.

Future research should continue investigating how material and relational investments jointly operate across different developmental periods and sociocultural contexts. Expanding relational indicators—such as parental warmth, monitoring, or emotion socialization—may clarify whether communication represents only one facet of relational engagement. Moreover, incorporating objective or multi-informant assessments of academic achievement may reduce reliance on subjective evaluations. Longitudinal models extending beyond three waves or employing advanced analytic strategies (e.g., random-intercept cross-lagged panel models or latent growth mediation models) could further illuminate how investment processes unfold over time. Finally, comparative research across Korean, East Asian, and Western contexts would help distinguish culturally specific patterns from universal mechanisms linking socioeconomic resources to youth development.

### Conclusion

In conclusion, this study provides longitudinal evidence that household income shapes adolescents’ academic performance primarily through material investments, particularly private educational expenditure, within the Korean educational context. The absence of a significant relational pathway highlights the distinctive ways in which socioeconomic resources are translated into academic opportunities in a highly institutionalized private education system. These findings further imply that the applicability of the traditional dual-pathway assumption of the Family Investment Model may vary across sociocultural contexts, with the Korean case demonstrating a more material-dominant configuration of investment processes. By applying a dual-pathway Family Investment Model to children in the later elementary years—a developmental period in which parental investments sharply intensify—this study extends prior FIM applications beyond early childhood and underscores the importance of developmental timing in understanding how socioeconomic disparities unfold. Although relational investments did not mediate academic outcomes, they may play a meaningful role in other developmental domains, pointing to the need for future research that examines multiple dimensions of parental engagement. Collectively, these findings contribute to a deeper understanding of socioeconomic inequalities in Korea and highlight the importance of continuing to investigate how distinct forms of parental investment shape educational trajectories across diverse cultural and educational settings. Taken together, the present study highlights the family material investment processes through which socioeconomic advantage is converted into unequal educational opportunities, underscoring the need to examine how resource-driven family dynamics reinforce income-linked academic disparities during critical developmental periods.

## Supporting information

S1 Table Robustness analysis using the original ordinal household income variable. (DOCX)

S2 Table Robustness analyses using weekday and weekend communication measures. (DOCX)
