# Supplementary material for: Household income and academic performance in Korean adolescents: A longitudinal test of dual investment pathways
Source: PLoS One. 2026 Jul 9;21(7):e0353476. doi: 10.1371/journal.pone.0353476 (PMC13349184; doi:10.1371/journal.pone.0353476)
Supplement: S1 Table — (DOCX) [file pone.0353476.s001.docx]

**Supplementary Materials** **S1**

Household Income and Academic Performance in Korean Adolescents: A Longitudinal Test of Dual Investment Pathways

**Robustness Analysis Using the Original Ordinal Household Income Variable**

To examine whether the findings were sensitive to the midpoint transformation of household income, a robustness analysis was conducted using the original 12-category ordinal household income variable. The identical longitudinal parallel mediation model was re-estimated using the original ordinal coding rather than the midpoint-coded continuous measure. As shown in Table S1, the pattern of results remained substantively unchanged across the two specifications. The material investment pathway remained statistically significant, whereas the relational investment pathway remained non-significant. In addition, the difference between the two indirect effects remained non-significant in both models. The magnitudes of the standardized path coefficients were highly similar across analyses, indicating that the substantive conclusions were not driven by the midpoint assignment procedure. These findings suggest that the observed associations between household income, family investments, and adolescents’ perceived academic performance were robust to alternative operationalizations of household income.

**Table S1** Comparison of Main Analysis and Robustness Analysis Using the Original Ordinal Household Income Variable

| Path | Main analysis (β) | Robustness analysis (β) |
| --- | --- | --- |
| Household income (W1) → Private educational expenditure (W2) | 0. 077*** | 0.078*** |
| Household income (W1) → Parent–child communication (W2) | 0.014 | 0.015 |
| Private educational expenditure (W2) → Perceived academic performance (W3) | 0.074** | 0.074** |
| Parent–child communication (W2) → Perceived academic performance (W3) | 0.085*** | 0.085*** |
| Household income (W1) → Perceived academic performance (W3) | 0.008 | 0.008 |
| Material indirect effect (HI → PEE → PAP) | 0.006** | 0.006** |
| Relational indirect effect (HI → PCC → PAP) | 0.001 | 0.001 |
| Difference between indirect effects (Material − Relational) | 0.002 | 0.002 |

*Note.* Values are standardized path coefficients. HI = household income; PEE = private educational expenditure; PCC = parent–child communication; PAP = perceived academic performance.

**p* < 0.05, ***p* < 0.01, ****p* < 0.001
