# Supplementary material for: Household income and academic performance in Korean adolescents: A longitudinal test of dual investment pathways
Source: PLoS One. 2026 Jul 9;21(7):e0353476. doi: 10.1371/journal.pone.0353476 (PMC13349184; doi:10.1371/journal.pone.0353476)
Supplement: S2 Table — (DOCX) [file pone.0353476.s002.docx]

**Supplementary Materials S2**

Household Income and Academic Performance in Korean Adolescents: A Longitudinal Test of Dual Investment Pathways

**Robustness Analysis Using Weekday and Weekend Communication Separately**

To examine whether the findings were sensitive to the operationalization of parent–child communication, additional robustness analyses were conducted using weekday and weekend communication separately rather than the composite communication measure used in the main analysis. The identical longitudinal parallel mediation model was re-estimated twice, first using weekday communication and then using weekend communication as the indicator of relational investment. As shown in Table S2, the substantive pattern of results remained unchanged across specifications. In both models, the material investment pathway remained statistically significant, whereas the relational investment pathway remained non-significant. Specifically, household income did not significantly predict either weekday or weekend communication, although both forms of communication were positively associated with perceived academic performance. In addition, the relational indirect effect remained non-significant in both models, whereas the material indirect effect remained statistically significant. These findings indicate that the study conclusions were not dependent on averaging weekday and weekend communication and were robust across alternative operationalizations of parent–child communication.

**Table S2** Comparison of Main Analysis and Robustness Analyses Using Weekday and Weekend Communication Separately

| Path | Main analysis (β) | Weekday Communication (β) | Weekend Communication (β) |
| --- | --- | --- | --- |
| Household income (W1) → Private educational expenditure (W2) | 0. 077*** | 0.077*** | 0.077*** |
| Household income (W1) → Parent–child communication (W2) | 0.014 | 0.013 | 0.015 |
| Private educational expenditure (W2) → Perceived academic performance (W3) | 0.074** | 0.076** | 0.071** |
| Parent–child communication (W2) → Perceived academic performance (W3) | 0.085*** | 0.083*** | 0.079*** |
| Household income (W1) → Perceived academic performance (W3) | 0.008 | 0.009 | 0.008 |
| Material indirect effect (HI → PEE → PAP) | 0.006** | 0.006** | 0.005** |
| Relational indirect effect (HI → PCC → PAP) | 0.001 | 0.001 | 0.001 |
| Difference between indirect effects (Material − Relational) | 0.002 | 0.002 | 0.001 |

*Note.* Values are standardized path coefficients. HI = household income; PEE = private educational expenditure; PCC = parent–child communication; PAP = perceived academic performance.

**p* < 0.05, ***p* < 0.01, ****p* < 0.001
